# Counteracting Cisplatin-Induced Testicular Damages by Natural Polyphenol Constituent Honokiol

**DOI:** 10.3390/antiox9080723

**Published:** 2020-08-09

**Authors:** Tse-En Wang, Yu-Hua Lai, Kai-Chien Yang, Sung-Jan Lin, Chih-Lin Chen, Pei-Shiue Tsai

**Affiliations:** 1 Department of Veterinary Medicine, National Taiwan University, No. 1, Sec. 4, Roosevelt Rd., Taipei 10617, Taiwan; f05629003@ntu.edu.tw (T.-E.W.); r05629012@ntu.edu.tw (Y.-H.L.); 2 Graduate Institute of Veterinary Medicine, National Taiwan University, Taipei 10617, Taiwan; 3 Research Center for Developmental Biology and Regenerative Medicine, National Taiwan University, Taipei 10617, Taiwan; kcyang@ntu.edu.tw (K.-C.Y.); drsjlin@ntu.edu.tw (S.-J.L.); 4 Institute of Pharmacology, College of Medicine, National Taiwan University, Taipei 10051, Taiwan; 5 Division of Cardiology, Department of Internal Medicine, College of Medicine, National Taiwan University, Taipei 10051, Taiwan; 6 Department of Biomedical Engineering, College of Medicine and College of Engineering, National Taiwan University, Taipei 10051, Taiwan; 7 Department of Dermatology, National Taiwan University Hospital and College of Medicine, Taipei 10002, Taiwan; 8 Department of Chemistry, National Taiwan University, Taipei 10617, Taiwan; chihlin0112538@gmail.com

**Keywords:** honokiol, cisplatin, oxidative stress, ER, TXNDC5, testis

## Abstract

Cisplatin, despite its anti-cancer ability, exhibits severe testicular toxicities when applied systemically. Due to its wide application in cancer treatment, reduction of its damages to normal tissue is an imminent clinical need. Here we evaluated the effects of honokiol, a natural lipophilic polyphenol compound, on cisplatin-induced testicular injury. We showed in-vitro and in-vivo that nanosome-encapsulated honokiol attenuated cisplatin-induced DNA oxidative stress by suppressing intracellular reactive oxygen species production and elevating gene expressions of mitochondrial antioxidation enzymes. Nanosome honokiol also mitigated endoplasmic reticulum stress through down regulation of Bip-ATF4-CHOP signaling pathway. Additionally, this natural polyphenol compound diminished cisplatin-induced DNA breaks and cellular apoptosis. The reduced type I collagen accumulation in the testis likely attributed from inhibition of TGFβ1, αSMA and ER protein TXNDC5 protein expression. The combinatorial beneficial effects better preserve spermatogenic layers and facilitate repopulation of sperm cells. Our study renders opportunity for re-introducing cisplatin to systemic anti-cancer therapy with reduced testicular toxicity and restored fertility.

## 1. Introduction

The main functions of testes are to produce spermatozoa and steroid hormones [1]. The physical barrier, called the blood-testis-barrier (BTB), formed by adjacent sertoli cells, restricts the passage of unwanted toxicants to the adluminal part of the seminiferous tubules, and ensures a proper environment for spermatogenesis [2]. Functional or structural disruption on BTB or sertoli cells will lead to sub/infertility in males. Cisplatin or cis-diamminedichloroplatinum(II), is an effective platinum anti-cancer compound; however, its usage is limited by accompanied severe side effects concurrently occurring in healthy tissues. Studies have shown that cisplatin binds not only to the nuclear DNA, but also to the mitochondria DNA (mtDNA) [3,4,5]. The formation of DNA adducts inhibits the cell cycle and results in cytotoxicity in both cancer and normal cells [4,5,6]. Apart from its notorious renal side effects, impairment of male reproductive functions and testicular damages were also reported [7,8,9]. With a cumulative dose of over 400 mg/m^2^, long-term infertility and irreversible failure on spermatogenesis were observed [10]. Earlier studies demonstrated that cisplatin-induced infertility resulted from its cytotoxicity on testicular germ cells, sertoli cells and leydig cells, a dose-dependent disruption of the seminiferous tubule epithelium and the increased apoptotic cells were also detected in the testes resulted in oligospermia and/or azoospermia [7,8,9,10].

Besides direct DNA damages, cisplatin also leads to excessive reactive oxygen species (ROS) production [11]. While balanced quantity of ROS maintains normal physiological functions of the spermatozoa, an excessive amount of ROS that exceeds cellular antioxidant ability will lead to oxidative stress (OS) [12]. Oxidative stress has been suggested as a potent cause of male infertility, as a high level of ROS in the seminal plasma was measured in 30% to 80% of in/sub-fertile men with impaired sperm motility, low sperm count and increased abnormal sperm morphology [13,14]. Cells prevent or ameliorate the deleterious effects of ROS by both enzymatic and non-enzymatic anti-oxidation systems. For the enzymatic anti-oxidation mechanism, superoxide dismutase (SOD), catalase, and glutathione peroxidase (GPx) are critical for testicular redox activity [15,16]. On the other hand, the non-enzymatic antioxidants, such as vitamin E, vitamin C, carotene, zinc and taurine can also act as radical scavenging agents or cofactors for non-enzymatic anti-oxidation activities [13,17]. Among non-enzymatic antioxidants, honokiol (HNK), a natural compound of polyphenol, has been shown to attenuate cisplatin-induced renal damages through the reduction of cellular oxidative stress and maintenance of the renal epithelium cytoskeleton [17,18]; moreover, HNK has been shown to reduce torsion-induced acute testicular injury via the reduction of excessive ROS production after ischemia-reperfusion injury [19]. Based on these studies, HNK is considered an effective scavenger for both superoxide and peroxyl radicals [20,21]. Although HNK might be effective on reducing cisplatin-induced OS, its lipophilic property restrains its bioavailability to tissue. To overcome this natural chemical solubility barrier of HNK, we encapsulated HNK into nanosized liposome (nanosome honokiol, nHNK) as described earlier to increase its bio-distribution and bio-availability [17,22]. This liposome-encapsulation approach has been shown to exhibit synergic and beneficial effects on anti-cancer ability when used in combination with cisplatin [23,24,25], which allows for the slow release of HNK into the body that prolongs its anti-oxidation effects in vivo.

In this study, we evaluated the effects of nHNK on cisplatin-induced testicular injury. The success of this study will not only allow for the understanding of HNK effects on cisplatin-induced chronic testicular injury, but can also lead to a potential clinical application for future use of HNK to reduce cisplatin-induced testicular toxicity in cisplatin-receiving cancer patients.

## 2. Materials and Methods

### 2.1. Chemicals, Reagents, Antibodies

Cis-Diamineplatinum(II) dichloride (Cisplatin, Cat. #479306, purity ≥ 99.9%) was purchased from Sigma Aldrich (St. Louis, MO, USA), 2-(4-hydroxy-3-prop-2-enyl-phenyl)-4-prop-2-enyl-phenol (Honokiol, Cat. #SLK S2310, purity: 99.81%) was obtained from Selleckchem (Houston, TX, USA). Other chemicals and reagents were acquired from Sigma unless otherwise stated. Mouse monoclonal anti-8 hydroxyguanosine (8-OHdG, #Ab62623), anti-type I collagen (#Ab6308), anti-glyceraldehyde 3 phosphate dehydrogenase (GADPH, #Ab9484), rabbit polyclonal anti-transforming growth factor beta 1 (TGFβ1, #Ab92486), goat polyclonal anti-alpha smooth muscle actin (αSMA, #Ab21027) antibodies were purchased from Abcam (Cambridge, UK); rabbit monoclonal anti-caspase 3 (#9665), rabbit polyclonal anti-binding immunoglobulin protein (BiP, #C50B12), mouse monoclonal anti-β actin (#3700), anti-X-box binging protein 1 splicing variant (XBP1s, #D2C1F) antibodies were obtained from Cell Signaling Technology Inc. (Danvers, MA, USA), rabbit polyclonal anti-activating transcription factor 4 (ATF4, #GTX101943), anti-activating transcription factor 6 (ATF6, #GTX104820) antibodies were purchased from GeneTex (Hsinchu, Taiwan), rabbit polyclonal anti-thioredoxin containing domain 5 (TXNDC5, #19834-1-AP), anti-CCAAT-enhancer-binding protein homologous protein (CHOP, #15204-1-AP) antibodies were obtained from Proteintech (Rosemont, IL, USA), rabbit monoclonal anti-phospho-Histone 2A. X (Ser139) (γ- H2A, clone JBW301, #05-636) was obtained from Millipore/Sigma (St. Louis, MO, USA). All secondary antibodies were purchased from Jackson ImmunoResearch Laboratories Inc. (West Grove, PA, USA).

### 2.2. Establishment of Cisplatin-Induced Chronic Testicular Injury Mouse Model

All in-vivo experiments were carried out under the approval and guidance of institutional animal care and use committee (IACUC) protocols (NTU107-EL-00155) at National Taiwan University, Taiwan and animal experiments were performed under daily supervision of certified veterinarians. Ten-week-old male institute of cancer research; Caesarean Derived-1 (ICR, CD1) mice were obtained from National Laboratory Animal Center, Taiwan and were acclimatized (3 mice/cage) for 1 week prior to experiments. The animal housing room was kept at a constant temperature (22–24 °C) with a 12 h alternating light-dark cycle. Animals were given water and standard mice lab chow (Oriental yeast, Tokyo, JP) ad libitum. Preparation of nanosized liposome-encapsulated honokiol (nHNK) were described earlier, qualitative and quantitative characterization of nNNK was carried out at the department of chemistry at National Taiwan University (NTU Mass Spectrometry Platform) and described earlier [17,22]. Animals were randomly allocated into 4 experimental groups (n = 10–12 for each group) and cisplatin-induced testicular injury mouse model was established as summarized in Figure S2A and described as follow. G1 (vehicle control): mice in this group were given three intraperinatal injections of 100 μL sterilized phosphate-buffered saline (PBS, red open circle), additional 100 μL of nanosized liposomes (vehicle control for HNK, blue open circle) was given at a three times/week interval for consecutive 6 weeks via tail vein injection (IV). G2 (nHNK, nanosome HNK group): besides sterilized PBS (as in G1), animals in this group received 5 mg/kg B.W. nHNK (blue closed circle, 100 μL volume) at a three times/week interval for 6 weeks. G3 (cisplatin injury group): chronic testicular injury was created by intraperinatal injections of 10 mg/kg B.W. cisplatin at week 0, 1 and 3 (red closed circle); moreover, mice in this group also received additional 100 μL of nHNK at the same intervals as mice in G2. G4 (cisplatin/nHNK, treatment group): mice in the treatment group were first injured by cisplatin as in G3, counteracting treatment was performed by injection of 5 mg/kg B.W. nHNK (blue closed circle, 100 μL volume) as indicated in the G2. Determination of nanosome honokiol tissue distribution in reproductive organs was carried out as described earlier [17,22].

### 2.3. Physical and Histological Evaluations

Body weight was measured bi-daily throughout the experiment. Blood was collected weekly through orbital sinus using a heparin coated capillary tube (Thermo Fisher Scientific, Waltham, MA, USA). Serum was separated from red blood cells by centrifugation at 2000 g for 10 min at 4 °C. Blood urea nitrogen (BUN) was analyzed by VITROS^®^ 350 Chemistry System (Ortho Clinical Diagnostics, Raritan, NJ, USA) at the clinical pathology unit at the School of Veterinary Medicine, National Taiwan University Teaching Hospital. At the end of the sixth week, mice were euthanized with CO_2_ followed by cervical dislocation. The weight of the testes and epididymides were measured. One side of the testes and epididymides were fixed in 10% buffered formalin for overnight on shaker and the other side of the tissues were flash-frozen immediately in liquid nitrogen and stored at −80 °C until used. Formalin-fixed testes and epididymides were subjected to standard paraffin-embedding procedures and the specimen was sectioned with a thickness of 5 μm. Before staining, sections were deparaffinized in xylene and rehydrated with 100–80% ethanol sequentially. Sections were stained with hematoxylin and eosin (H&E) for general histological evaluations. Testicular and epididymal sections were examined and evaluated in random order under blindfold conditions with Olympus IX83 microscopy (Tokyo, Japan). Structural changes of the seminiferous tubules were semi-quantification using a self-designed testis damage scoring system (Table 1). In each group, testicular sections were classified by the degree of damages ranging from 0 to 4 (no damage to severe). With this scoring system, the seminiferous tubules with >3, 2, 1 and 0 layer(s) of spermatogenic cells present were scored in a reciprocal point of 0, 1, 2, and 3 points, respectively. If multinucleated giant cells were observed in the lumen (indication of necrosis), one additional point will be added. Therefore, the higher point represents a more severe cellular loss in the seminiferous tubules. Total points within the same experimental condition were added up and were divided by the numbers of the lumen examined to obtain the average points/lumen of each experimental condition. For general sperm production evaluation, the amount of sperm cells present in the caudal epididymal lumen was assessed and expressed as the percentage of the cauda epididymal lumen with the presence of sperm cells. Ten random H&E stained images of both testis and epididymis from each group (total 40 images were reviewed) were further quantified using Olympus IX83 microscopy.

### 2.4. Cell Culture

Mouse sertoli cell line, TM4 was obtained from ATCC (# CRL-1715^™^, Manassas, VA, USA). Cells were cultured in Dulbecco’s Modified Eagle Medium/Nutrient Mixture F-12 (DMEM/F12, Gibco, Waltham, MA, USA) supplemented with 5% horse serum, 2.5% fetal bovine serum and 1% penicillin-streptomycin-amphotericin B (Gibco) at 37 °C in a humidified atmosphere with 5% CO_2_. For whole cell lysate, after designed treatments, cells were rinsed three times with ice-cold dulbecco’s phosphate-buffered saline (DPBS, Gibco) and subsequently scraped into radioimmunoprecipitation assay buffer (RIPA) lysis buffer (Boston BioProducts, Boston, MA, USA) supplemented with ethylenediaminetetraacetic acid (EDTA) free protease inhibitors (Roche, Mannheim, Germany). Cells were lysed on ice for 15 min and sonication for 1 min in ice-cold water bath. The lysates were centrifuged at 10,000 *g* for 10 min at 4 °C to remove cellular debris. Protein quantification was carried out with bicinchoninic acid (BCA) protein assay kit (Pierce, Wilton, IL, USA), and cells lysates were stored at −20 °C for later use.

### 2.5. Cell Viability Assay (MTT Assay)

To determine the cytotoxicity of cisplatin and HNK, 3-(4,5-Dimethylthiazol-2-yl)-2,5-Diphenyltetrazolium Bromide (MTT) (Sigma Aldrich) assay was carried out. MTT stock (5 mg/mL) was prepared by dissolving MTT powder into PBS and stored at −20 °C for later use. Cells were seeded in 96- well plates at a density of 2.5 × 10^4^ cells per well and grew for 24 h. Cells were then serum-starved for 2 h prior to cisplatin or HNK treatments. Four hours before the end of treatments, MTT stock solution was added into each well (10 μL/well, final working concentration of 0.45 mg/mL) and incubated at 37 °C for 4 h. Unbound MTT was removed from the supernatant, and the formed crystals were dissolved in 100 μL of dimethyl sulfoxide (DMSO) followed by additional incubation of 1.5 h at room temperature (RT). Optical density (OD) was measured at a wavelength of 570 nm, and background values were measured at 650 nm with a SpextraMax M5 microplate reader (Molecular Devices, San Jose, CA, USA).

### 2.6. Measurement of Intracellular ROS Production

Generation of cellular ROS was monitored by membrane-permeable dye 2′,7′-dichlorofluorescein diacetate (DCFH-DA). Cells were seeded in 12- well plates at a density of 10^6^ cells per well and grown for 24 h. Cells were serum-starved for 2 h prior to cisplatin or HNK treatments. After the treatments, cells were incubated with phenol red-free DMEM/F12 contained 25 μM DCFH-DA at 37 °C for 30 min at dark. After washed with iced-cold PBS and analyzed with FACScalibur flow cytometer (Becton and Dickinson, Pharmingen, Franklin Lakes, NJ, USA). Data were further processed and analyzed with the BD CellQuest Pro Software. To further confirm and determine cisplatin-induced ROS production, cellular reactive oxygen species detection assay kit (deep red fluorescence) (Abcam, #186029) was used. 96-wells was first coated with matrigel (matrigel: DPBS = 1:10) and incubate at 37 °C for 30 min before seeding of TM4 cells at a density of 10^4^ cells/well for 24 h. After the required treatments, supernatant was removed and 100 μL of cell-permeable ROS deep red sensor was added into each well as suggested by manufactory instruction. Fluorescence intensity reflecting the amount of intracellular ROS was monitored at Ex/Em = 650/675 nm using SpextraMax M5 microplate reader (Molecular Devices, San Jose, CA, USA).

### 2.7. Antioxidant Enzyme Assays

To examine the in-vivo effect of nHNK on anti-oxidation enzyme activities, total anti-oxidant enzyme activity was determined with the OxiSelect™ Total Antioxidant Capacity Assay kit (TAC assay, Cell Biolabs, Inc., San Diego, CA, USA. Testes from mice of different treatments were homogenized with ice-cold homogenizing buffer (1 mM EDTA, 20 mM Tris-HCl, 20 mM HEPES, 250 mM sucrose and protease inhibitor, pH 7.5). All assays were performed according to manufacturer’s instructions and end-point measurements were performed and compared accordingly.

### 2.8. Next Generation Sequencing (RNA-seq) and Library Preparation for Transcriptome Sequencing

A total amount of 1 μL total RNA per sample was used as input material for the RNA sample preparations. Sequencing libraries were generated using KAPA mRNA HyperPrep Kit (KAPA Biosystems, Roche, Basel, Switzerland) following manufacturer’s recommendations and index codes were added to attribute sequences to each sample. Briefly, mRNA was purified from total RNA using magnetic oligo-dT beads. Captured mRNA was fragmented by incubating at a high temperature in the presence of magnesium in KAPA Fragment, Prime and Elute Buffer (1×). First strand cDNA was synthesized using random hexamer priming. Combined 2nd strand synthesis and A-tailing, which converts the cDNA:RNA hybrid to double-stranded cDNA (dscDNA), incorporated dUTP into the second cDNA strand, and added dAMP to the 3′ ends of the resulting dscDNA. dsDNA adapter with 3′dTMP overhangs were ligated to library insert fragments to generate the library fragments carrying the adapters. In order to select cDNA fragments of preferentially 300~400 bp in length, the library fragments were purified with KAPA Pure Beads system (KAPA Biosystems, Roche, Basel, Switzerland). The library carrying appropriate adapter sequences at both ends was amplified using KAPA HiFi HotStart ReadyMix (KAPA Biosystems, Roche, Basel, Switzerland) along with library amplification primers. The strand marked with dUTP in not amplified, allowing strand-specific sequencing. At last, PCR products were purified using KAPA Pure Beads system and the library quality was assessed on the Qsep 100 DNA/RNA Analyzer (BiOptic Inc., Taiwan).

The original data obtained by high-throughput sequencing (Illumina NovaSeq 6000 platform) were transformed into raw sequenced reads by CASAVA base calling and stored in FASTQ format. FastQC and MultiQC [26] were used to check fastq files for quality. The obtained raw paired-end reads were filtered by Trimmomatic (v0.38) [27] to discard low-quality reads, trim adaptor sequences, and eliminate poor-quality bases with the following parameters: LEADING:3 TRAILING:3 SLIDINGWINDOW:4:15 MINLEN:30. The obtained high-quality data (clean reads) was used for subsequent analysis. FeatureCounts (v1.6.0) was used to count the reads numbers mapped to individual genes [28]. For gene expression, the “Trimmed Mean of M-values” normalization (TMM) was performed DEGseq (v1.36.1) [29] without biological duplicate and the “Relative Log Expression” normalization (RLE) was performed using DESeq2 (v1.22.1) [30,31] with biological duplicate. Differentially expressed genes (DEGs) analysis of two conditions was performed in R using DEGseq (without biological replicate) and DESeq2 (with biological replicate), which based on negative binomial distribution and Poisson distribution model, respectively [32,33,34].

### 2.9. Computer-Assisted Sperm Analysis (CASA)

To evaluate the effects of cisplatin injury and nHNK treatment on sperm motility-related parameters, UltiMate computer-assisted sperm analysis system (CASA, Hamilton Thorne Inc., Beverly, MA, USA) was used as described by Broekhuijse et al. [35]. Definition of motility (%), progressive motility (%), velocity average path (VAP, µm/s), velocity straight line (VSL, µm/s), velocity curvilinear (VCL, µm/s), amplitude of lateral head displacement (ALH, µm), beat frequency cross (BCF, Hz), straightness (STR) and linearity (LIN) were followed by default recommendations from Hamilton Thorne Inc. for mouse spermatozoa. Sperm samples from 4 experimental conditions were subjected to analysis using a standardized Leja 2-chamber counting slide (Leja Products B.V., Nieuw Vennep, the Netherlands), image capture was set to 60 frames/sec; a total 45 frames were recorded per examination field. By the use of automated stage, 5 independent microscopic fields (a total 225 frames were taken per experimental sample) were analyzed and at least 6 independent biological repeats were performed for each experimental condition; mean value and standard deviation (SD) were calculated accordingly.

### 2.10. Indirect Immunofluorescence (IFA) and Immunohistochemistry (IHC) Staining

Indirect immunofluorescent staining was carried out as described earlier [36]. Tissue sections were deparaffinized with 100% xylene and rehydrated with 100–80% ethanol. Antigen retrieval was carried out by heating tissue sections in 10 mM citrate buffer (pH 8.0) at 95 °C and 104 °C for 5 min at each temperature. After blocked with 1% bovine serum albumin (BSA) for 60 min at RT, tissue sections were further permeabilized with 0.1% Triton-X 100 at RT for 5 min. Anti- 8-OHdG, anti-gamma phospho-Histone 2A. X (γ-H2A) and anti-collagen type I antibodies were used at a dilution of 1:1000, 1:100 and 1:250, respectively, and incubated for overnight at 4 °C. For IFA, sections were subsequently incubated with goat anti-mouse or goat anti-rabbit Alexa-594 (1:150 diluted with 1% BSA) for 1.5 h at RT. Nuclei were counterstained with 4′,6-diamidino-2-phenylindole (DAPI) (Vectashield H-1200, Vector Laboratories, Peterborough, UK) and slides were sealed with nail polish. For IHC, Dako REAL^TM^ EnVision^TM^ Detection System (Peroxidase/DAB+ Rabbit/Mouse, Glostrup, Denmark) was used according to manufacturer’s instructions. As for negative controls, each immunoreaction was accompanied by a reaction omitting the primary antibody. All samples were evaluated with Olympus IX83 epifluorescent microscopy. All images were subsequently analyzed with either ImageJ (NIH; http://rsb.info.nih.gov/ij/) or CellSens software (Tokyo, Japan). When necessary, background subtraction and contrast/brightness enhancement (up to ~20% enhancement using the maximum slider in both software) were performed identically for all images in the same sets of experiment.

### 2.11. Terminal Deoxynucleotidyl Transferase-Mediated dUTP-Biotin Nick End Labeling (TUNEL) Assay

To detect the level of apoptosis in the testis, paraffin-embedded tissue sections were proceeded for TUNEL assay using a DeadEnd™ Fluorometric TUNEL System (Promega, Madison, WI, USA) according to manufacturer’s instruction with nuclei counterstained with DAPI. To assess the number of apoptotic cells in the testis, 10 random images from each experimental group were taken under 200× magnification using Olympus IX83 microscopy and positive signals were quantified by CellSens software. The number of TUNEL positive cells was divided by total number of cells (indicated by DAPI) in each image frame to obtain the percentage of TUNEL positive cells per examination frame, an average percentage of 10 images were further calculated.

### 2.12. Immuno-Blotting

Equivalent amount of protein extract (μg) was resuspended with an appropriate volume of lithium dodecyl sulfate (LDS) loading buffer (NuPAGE™, Thermo Fisher Scientific) in the presence of reducing agent (50 mM dithiothreitol [DTT]). Samples were heated in a 100 °C-dry bath for 10 min and air cooled to RT before loading on gels. Bio-Rad Mini-PROTEIN^®^ electrophoresis system was used (Bio-Rad Laboratories Ltd., Hertfordshire, UK) and standard manufactory protocol was followed. Proteins were separated by 10% sodium dodecyl sulfate- polyacrylamide gel (SDS-PAGE, gradient T-Pro EZ Gel Solution, T-Pro Biotechnology, New Taipei County, Taiwan) and wet-blotted onto a Polyvinylidene difluoride (PVDF) membrane (Immobilon-P, Millipore, Burlington, MA, USA). After blocking for 1 h with blocking buffer Tri-buffered saline-Triton X100 (5 mM Tris, 250 mM sucrose, pH 7.4 with 0.05% *v/v* Tween-20 [TBST], supplemented with 5% milk powder) at RT, blots were incubated with anti-BiP antibody (1:1000 dilution), anti-ATF4 antibody (1:1000 dilution), anti-CHOP antibody (1:1000 dilution), anti-ATF6 antibody (1:1000 dilution), anti-XBP1s antibody (1:1000 dilution), anti-β actin antibody (1:10000 dilution), anti-TXNDC5 antibody (1:1000 dilution), anti-caspase 3 antibody (1:1000 dilution), anti-TGFβ1 antibody (1:500 dilution), anti-GADPH antibody (1:10000 dilution), anti-αSMA antibody (1:1000 dilution) or anti-collagen type I antibody (1:1000 dilution) at 4 °C for overnight. After three times washing in TBST, secondary antibody was subsequently added and blots were incubated at RT for another 1h. After rinsing with TBST, specific protein signal was visualized by chemiluminescence (Merck, Ltd., Kenilworth, NJ, USA) and detected under ChemiDoc™ XRS+ system (Bio-Rad Laboratories, Hercules, CA, USA). The relative intensity of each band was determined using ImageJ software. When necessary, blots were stripped with stripping buffer (Thermo Fisher Scientific) and re-probed for other proteins of interests.

### 2.13. Sample Preparation and Analysis on High Performance Liquid Chromatography (HPLC)-MS/MS

Samples were prewashed with sterilized PBS to remove blood on the surface of the organs. YSZ grinding media (EZEAG0100, Oriental Cera Tec., Inc., Taiwan) and a defined volume of methanol were added to each sample after weighting. A mini-bead beater (BioSpec Product, Inc., Bartlesville, OK, USA) was used to homogenize the organs for 90 s in a cold room at 7 °C. The resulting homogenates were centrifuged at 4 °C for 5 min, and the supernatants were collected. The residual samples were extracted again by the same process, and the supernatants were combined and preserved in −80 °C prior to HPLC-MS/MS analysis. For HPLC-MS/MS analysis, 50 μL of the extract was spiked with 50 μL of the internal standard solution and analyzed (Figure S5B). A calibration curve was measured prior to quantification of HNK in the mouse organs using the same HPLC-MS/MS method. A series of HNK solutions (0.5, 1.0, 5.0, 10.0, 25.0, and 50.0 ppb; water/methanol = 1:1, *v/v*) was first prepared. A stock solution of 1,1-Bis(4-hydroxypheny)-cyclohexane (1mg/1ml) dissolved in DMF/methanol (1:49, *v/v*) was diluted with water/methanol (1:1, *v/v*) to 100 ppm as an internal standard solution. Then 100 μL of each honokiol calibrating solution was spiked with an equal volume of the internal standard solution and analyzed with HPLC-MS/MS.

HPLC-MS/MS analysis was performed with a C-18 reverse phase column (Atlantis T3, 3 μm, 21 × 100 mm, Waters, Milford, MA, USA) coupled with an ExionLC AC and with an AB SCIEX Triple Quad 5500 equipped with an electrospray ionization source. The chromatographic separation was performed with an isocratic gradient elution of 0.1% formic acid in water/acetonitrile (20:80, *v/v*) in a flow rate of 0.5 mL/min at 40 °C for 5 min. The injection volume was 5 μL. Negative ion mode of ESI with ion spray voltage at 4.5 kV and temperature at 600 °C was chosen for honokiol profiling. The nebulizer gas and heater gas were set at 55 and 60 psi, respectively. The software Sciex analyst (version 1.6.2) were used to acquired spectra and to process data. Honokiol quantification in each tissue organ of different time points was performed by calculating peak area ratio of the ion fragment with the highest peak intensity and the internal standard using MultiQuantTM 3.0.2.

### 2.14. Statistical Analysis

All values are presented as mean ± standard deviation (SD). One-way analysis of variance (ANOVA) with Tukey’s multiple comparisons test or two-tail Student’s t-test was used to evaluate statistical differences via GraphPad Prism (GraphPad Software, San Diego, CA, USA). Comparisons of testis damage score was performed with non-parametric Kruskal-Wallis test with Dunnett’s Multiple Comparison. Differences were considered statistically significance at *p* value < 0.05.

## 3. Results

### 3.1. Honokiol Attenuated Cisplatin-Induced Intracellular ROS Production in Mouse Sertoli Cells

**Figure 1:**
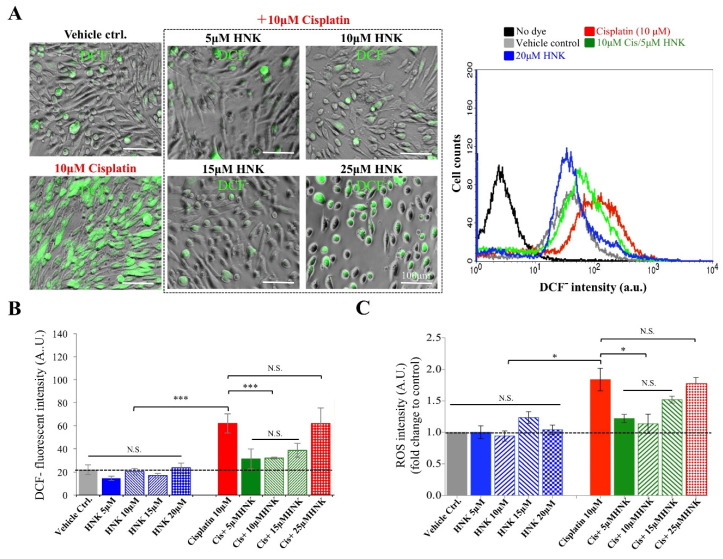
Honokiol effects on cisplatin-induced intracellular reactive oxygen species (ROS) production in mouse sertoli cells. (**A**) Mouse sertoli cells were stained with DCFH-DA to evaluate the amount of intracellular ROS. Cisplatin-induced intracellular ROS production was observed by indirect fluorescent microscopy and DCF- positive cells (in green) showed mostly in round shape indicated unhealthy status. Co-incubation of 5–15 μM HNK reduced intracellular ROS production. Flow cytometry analysis (right panel) confirmed that cisplatin-induced overproduction of intracellular ROS (red line) was reduced by honokiol (green line) in vitro. (**B**) Two independent ROS assays showed while 10 μM cisplatin caused excessive ROS production, HNK alone did not induced overproduction of ROS. Co-incubation of 5–15 μM HNK significantly reduced intracellular ROS production. However, when >25 μM HNK was used in combination with 10 μM Cisplatin (patterned red bars), a detrimental effect was detected. One-way analysis of variance (ANOVA) with Tukey’s multiple comparisons test was used and statistical difference at *p* < 0.05, N.S.: no statistical different. *** *p* < 0.001; * *p* < 0.05; Representative images were shown.

Excessive ROS production has been shown to associate with cisplatin-induced cell death. To demonstrate ROS reducing and anti-oxidation ability of HNK, membrane permeable 2′,7′-dichlorofluorescein diacetate (DCFH-DA), a compound that can actively reacts with multiple ROS species [37] was used. We showed an increased cytotoxicity of cisplatin when concentration was ≥15 μM; however, this was not observed for HNK up to 25 μM (Figure S1). A synergistic cytotoxicity of cisplatin/HNK combination was detected when HNK concentration exceeded 25 μM (red bar, Figure S1) suggested caution is required when cisplatin and HNK were used in combination. Based on cell viability tests, we thereafter applied 10 μM cisplatin with various HNK concentrations (0–20 μM) in our following in-vitro tests. We observed that cisplatin induced excessive intracellular ROS production in mouse sertoli cells (evidenced by increased DCF- signals, Figure 1A), and DCF- positive cells showed round-shaped (representing dead or unhealthy cells) morphology rather than well-spread adherent cells (Figure 1A). When HNK was used in combination, significant reduction of DCF- signals was detected. This was further supported by flow cytometry analysis that application of 5μM HNK can already reduce cisplatin-induced ROS (Figure 1A, right panel, compared red and green lines). Quantitative analyses from two independent assays demonstrated that HNK alone did not elicit excessive intracellular ROS (Figure 1B,C, blue bars), and cisplatin-treated cells, when co-incubated with HNK under 25 μM showed significant reduction of intracellular ROS (Figure 1B,C, green bars).

### 3.2. Cisplatin-Induced Testicular Damage and Reduction on Sperm Production Were Abated by nHNK

**Figure 2:**
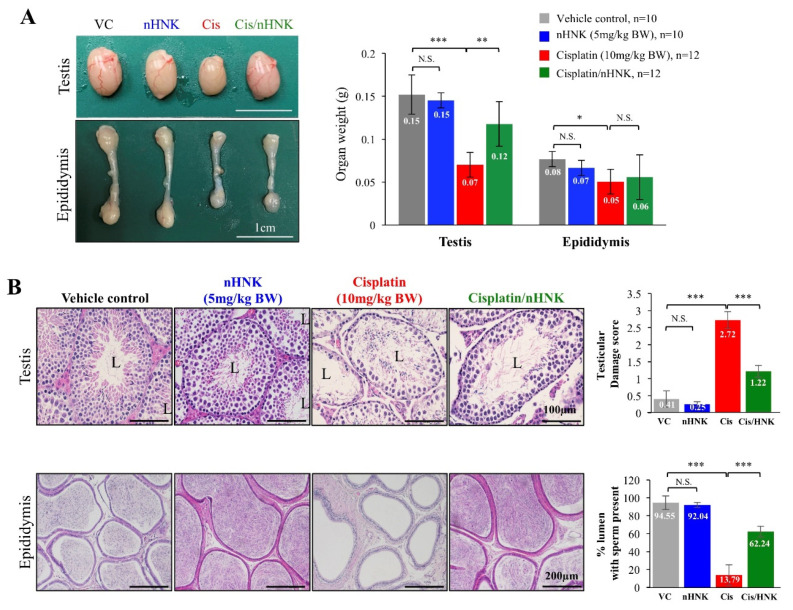
The effects of nanoparticulated honokiol on cisplatin-induced testicular damages and sperm production. (**A**) In contrast to control, nHNK and cisplatin/nHNK group, testis and epididymis from cisplatin-treated animals were smaller in size and pale at appearance. An apparent recovery was detected in the testis, but not epididymis of nHNK-treated animals (**B**) Histology examination on testicular tissue showed multiple spermatogenic layers in control and nHNK animals. Reduced seminiferous tubule epithelium layer and disrupted testicular structure were observed in cisplatin-treated mice, whereas a partial recovery of testicular epithelium layer was observed in cisplatin/nHNK-treated mice. Testicular damage scoring was performed using non-parametric Kruskal-Wallis test with Dunnett’s Multiple Comparison. General sperm production was evaluated by checking the presence of spermatozoa in the lumen of cauda epididymis. A significant decrease of sperm production was evidenced by low% of lumen contained spermatozoa within; a significant restoration of spermatogenesis with increased amount of epididymal sperm cells was observed when nHNK was given. VC: vehicle control; nHNK: nanosome honokiol alone; Cis: 10 mg/kg B.W. cisplatin administration; Cis/nHNK: nHNK treatment group. One-way analysis of variance (ANOVA) with Tukey’s multiple comparisons test was used for group comparisons and statistical difference at *p* < 0.05, N.S.: no statistical different. *** *p* < 0.001; ** *p* < 0.01; * *p* < 0.05; Representative images were shown.

Cisplatin is known to cause renal dysfunctions. To validate the success of our mouse model (Figure S2A), we used as previously described, body weight and renal physiological parameters as indications [17]. We showed earlier that 5 mg/kg B.W. is the lowest effective concentration of nHNK to provide protective outcomes against cisplatin-induced renal damages [17], in agreement with that, cisplatin-receiving mice showed significant reduction on body weight (−15%) with increased serum blood urine nitrogen (BUN, +198%) (Figure S2B) indicated successful establishment of our mouse model. From In Vivo data, we observed a significant reduction on the weight and the size of both testes and epididymes in cisplatin-treated animals. Moreover, a pale appearance was observed for cisplatin-treated testes and epididymes (Figure 2A). In contrast, when nHNK was used, above-mentioned changes were attenuated (Figure 2A).

Unlike in control and nHNK-treated testes showed normal testicular structure with 4–5 spermatogenic layers, histological evaluation on cisplatin-treated testis showed a disrupted testicular architecture, loss of spermatogenic cell, severe sloughing of cellular materials in the lumens and were scored significantly higher in testicular damage scoring system (2.7 ± 0.2, Figure 2B, red bar). Although decreased number of differentiated germinal cells was also observed in the lumens of cisplatin/nHNK- treated animals, the damages were significantly less severe (1.2 ± 0.2, Figure 2B, green bar). Besides testicular structure, general sperm production was also affected. While >90% of the lumens from both control (94.6 ± 0.1) and nHNK (92.0 ± 0.0) groups contained sperm cells within, we observed only 13.8 ± 0.1% of the lumen from cisplatin-treated mice contained sperm cells. In sharp contrast, when nHNK was given to cisplatin-treated animals, not only testicular structure was partially restored, a significant recovery (62.2 ± 0.9%) on sperm production was also observed (Figure 2B).

### 3.3. Cisplatin-Induced Low Sperm Count, Decreased Motility and Defected Acrosome Reaction Ability Were Partially Restored after nHNK Treatment

To assess whether restoration of sperm production also reflect on sperm quality, we carried out computer assisted sperm analysis (CASA) and acrosome reaction assay to evaluate general sperm quality. In line with observations in Figure 2B, cisplatin greatly reduced sperm concentration (12.19 × 10^6^ and 5.43 × 10^6^ sperm/mL in control and cisplatin- treated mice, respectively). Moreover, decreased sperm motility (85.5% and 60.7% in control and cisplatin- treated mice, respectively) and progressive motility (49.5% and 33.9% in control and cisplatin- treated mice, respectively) were also measured. However, in nHNK-treated mice, not only sperm concentration was restored (9.77 × 10^6^ sperm/mL), both sperm motility (84.1%), progressive motility (46.2%) and straight line velocity (17.92 mm/seg) were also improved (Figure S3A). When PNA was used to evaluate sperm acrosome integrity, we observed that while 53%, 32% and 31.5% of the sperm cells from control, nHNK and cisplatin/nHNK were able to respond to calcium ionophore- induced acrosome reaction that merely 2% of the sperm cells from cisplatin-treated mice were responding to the same stimulation (Figure S3B).

### 3.4. Nanosome Honokiol Reduced DNA Oxidative Stress in the Testis

**Figure 3:**
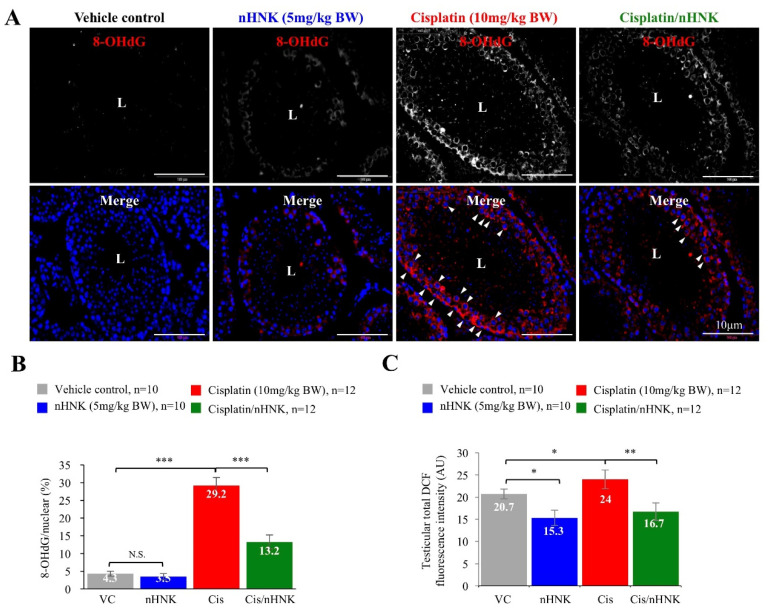
The effects of nanosome honokiol on cisplatin-induced DNA oxidative stress. (**A**) DNA oxidative stress marker 8-OHdG signals were observed mostly at the basal region of the seminiferous tubules (indicated with arrows) in the testis of cisplatin- injured animals while minimal to reduced 8-OHdG signals were detected in the control, nHNK or cisplatin/nHNK treatment groups. (**B**) Quantification analysis supported significant increase of 8-OHdG in the testis of cisplatin-injured mice, and nHNK efficiently reduced DNA oxidative stress marker 8-OHdG. (**C**) DCFH-DA analysis was used to measure intracellular ROS production in the testicular cells obtained from the animals. Increased DCF- signal was detected upon cisplatin treatment, both nHNK alone and cisplatin/nHNK co-treatment significantly reduced DCF-signals. VC: vehicle control; nHNK: nanosome honokiol alone; Cis: 10 mg/kg B.W. cisplatin administration; Cis/nHNK: nHNK treatment group. One-way analysis of variance (ANOVA) with Tukey’s multiple comparisons test was used and statistical difference at *p* < 0.05, N.S.: no statistical different. *** *p* < 0.001; ** *p* < 0.01; * *p* < 0.05; Representative images were shown. L: lumen.

To examine whether nHNK attenuation of testicular injury was associated with its anti-oxidation ability, we evaluated the presence and the amount of DNA oxidative stress marker 8 hydroxyguanosine (8-OHdG) in the testicular tissue. We observed increased 8-OHdG at the basal region of the seminiferous tubules (Figure 3A, indicated with arrowheads). Quantitative analysis further confirmed cisplatin induced DNA oxidative damages as 29.2% of the testicular tissue showed positive signals for 8-OHdG (6.79-fold increase when compared with control mice). In contrast, a 45% reduction of 8-OHdG signal was detected when nHNK was used, indicating nHNK protected testis from cisplatin-induced oxidative damages in vivo (Figure 3B, from 29.2% in cisplatin group to 13.2% in Cis/nHNK group). This observation was also evidenced from testicular ROS measurement using membrane permeable DCFH-DA. Cisplatin led to higher amount of DCF- signal in the testis and nHNK treatment significantly reduced DCF- signal in the testis (Figure 3C).

### 3.5. Nanosome Honokiol Reduced Endoplasmic Reticulum Stress in the Testes

**Figure 4:**
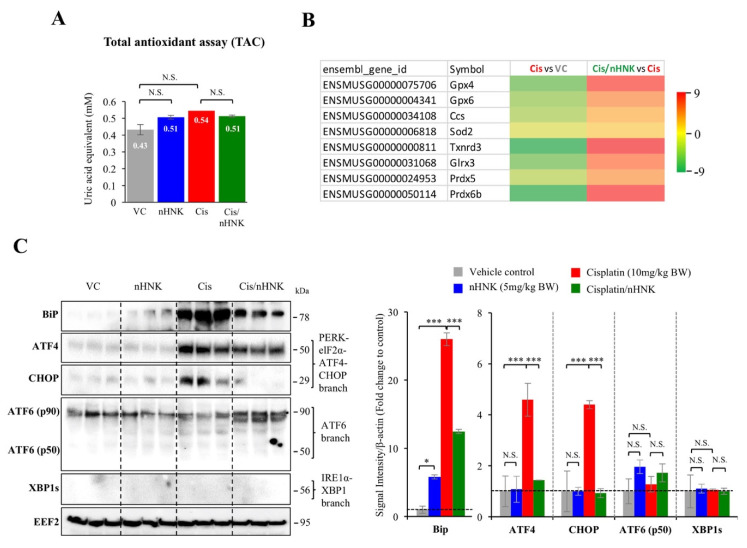
The effects of nanosome honokiol on mitochondria enzymatic machinery and endoplasmic reticulum-associated stress. (**A**) Total antioxidant assay measured total cellular and mitochondria enzymatic antioxidant ability showed no statistical differences between groups. (**B**) RNAseq analysis showed down regulation of mitochondrial anti-oxidation enzymes glutathione peroxidase (Gpx), thioredoxin reductase (Txnrd), glutaredoxin (Glrx), peroxiredoxin (Prdx) upon cisplatin treatment and nHNKwas able to upregulated gene expressions of these anti-oxidation enzymes. VC: vehicle control; nHNK: nanosome honokiol alone; Cis: 10 mg/kg B.W. cisplatin administration; Cis/nHNK: nHNK treatment group. At least 3 independent experiments or animals were performed or used. One-way analysis of variance (ANOVA) with Tukey’s multiple comparisons test was used and statistical difference at *p* < 0.05, N.S.: no statistical different. *** *p* < 0.001; * *p* < 0.05.

To investigate the mechanism of nHNK effect, total antioxidant assay (TAC) which evaluate total cellular antioxidant ability (TAC) was performed. Moreover, RNAseq analysis using testicular samples from in-vivo experiments was also performed to evaluate mitochondrial antioxidation enzyme activities. Unexpectedly, unlike our earlier report in the kidney [17,18], TAC assay did not show significant changes between groups (Figure 4A); however, from RNAseq analysis, mitochondrial antioxidant enzymes including glutathione peroxidase (Gpx), thioredoxin reductase (Txnrd), glutaredoxin (Glrx), peroxiredoxin (Prdx) were downregulated upon cisplatin treatment, and were upregulated when nHNK was given as treatment (Figure 4B). These data suggested cisplatin and nHNK altered mitochondrial enzymatic machinery in the testis. An earlier study by Wang et.al showed platinum-induced cell apoptosis might be mediated by endoplasmic reticulum (ER)-stress [5], we next examined whether nHNK reduction on testicular oxidative damages was related to ER stress. We showed upstream ER stress marker protein binding immunoglobulin protein (Bip) was significantly increased in cisplatin-injured mice. Moreover, downstream activating transcription factor 4 (ATF4) and CCAAT-enhancer-binding protein homologous protein (CHOP) proteins but not activating transcription factor 6 (ATF6) or X-box binging protein 1 splicing variant (XBP1s) were up-regulated in the testis of cisplatin-injured mice (Figure 4C, Figure S4). Quantitative analyses showed significant decrease on two ER stress marker proteins ATF4, CHOP, but not ATF6 or XBP1 when nHNK was given to cisplatin-injured mice (Figure 4C, right panel). These data indicated that nHNK mitigated cisplatin-induced testicular ER stress via specific Bip-ATF4-CHOP signaling pathway.

### 3.6. nHNK Attenuation of Testicular Apoptosis was Associated with Reduced DNA Breaks

**Figure 5:**
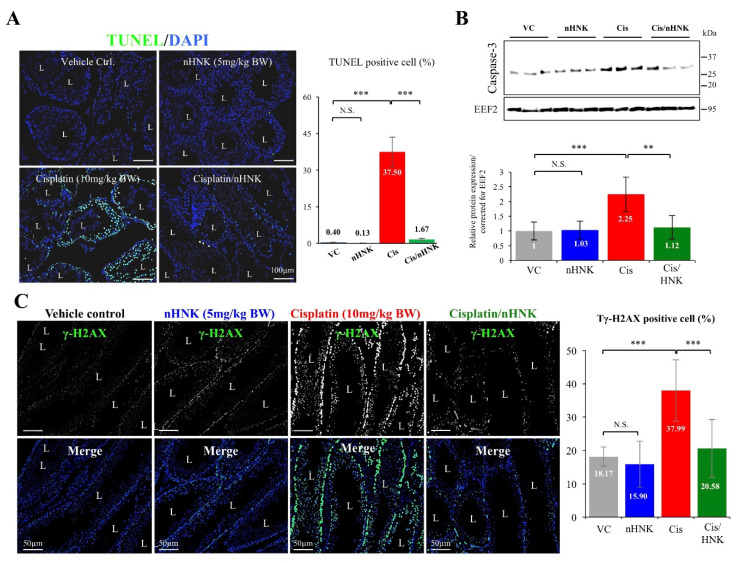
Nanosome honokiol attenuated cellular apoptosis by reducing DNA breaks in the testis. (**A**) TUNEL assay showed increased number of apoptotic cells (in green) in the testis of cisplatin-injured mice while nHNK treatment significantly reduced cellular apoptosis. (**B**) Apoptosis marker protein caspase 3 increased 2.25-fold in cisplatin-treated mice. A reduction of caspase 3 protein expression was detected in the testis of nHNK-treated mice. (**C**) Gamma- H2A staining was used to assess the level of DNA breaks in the testis; a significant increase of γ-H2AX signal was detected in the nuclei of cisplatin-treated testis and nHNK treatment significantly reduced the% of γ-H2AX positive cells. One-way analysis of variance (ANOVA) with Tukey’s multiple comparisons test was used and statistical difference at *p* < 0.05, N.S.: no statistical different. *** *p* < 0.001; ** *p* < 0.01. Representative images were shown.

The formation of DNA adducts by cisplatin is known to inhibit cell cycle and results in cellular apoptosis [6,11]. To evaluate cisplatin-induced apoptosis and nHNK effects, terminal deoxynucleotidyl transferase-mediated dUTP-biotin nick end labeling (TUNEL) assay was performed. While a minimal number of TUNEL positive cells were detected in control (0.4%) and nHNK (0.1%) groups, significant increase of apoptotic cells (37.5%) was observed in the testis of cisplatin-treated animals. In a sharp contrast, nHNK significantly ameliorated cisplatin-induced apoptosis in the testis (Figure 5A). When apoptosis marker caspase-3 was further examined, in consistence with TUNEL assay, we showed cisplatin led to up-regulation of caspase-3 protein expression in the testis and nHNK treatment resulted in the reduction of caspase-3 protein expression (Figure 5B, Figure S4), although we did not detect the emergence of cleaved caspase 3 in the testicular homogenates, but significant increase of full length caspase 3 protein expression upon cisplatin injury may suggested the involvement of caspase 3 signaling pathway in cisplatin-induced testicular damages as we showed earlier in the kidney. These above-mentioned results were likely attributed from the increased DNA breaks (indicated by positive γ-H2AX signals) in the testis of cisplatin-treated mice, and the attenuated outcomes from nHNK treatment (Figure 5C, 18%, 16%, 38% and 21% for control, nHNK, cisplatin and cisplatin/nHNK group, respectively).

### 3.7. Cisplatin-Induced Loss of Spermatids Was Partially Restored upon nHNK Treatment

**Figure 6:**
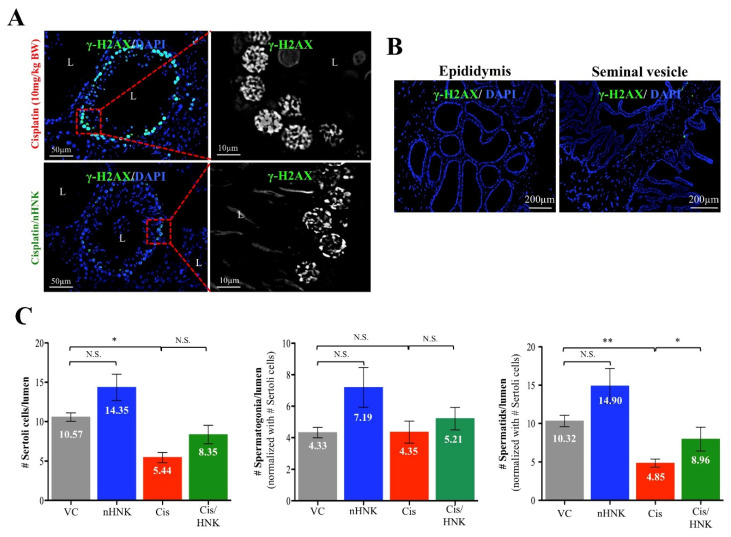
The effects of cisplatin and nHNK on spermatogenic cells. (**A**) Enlarged images showed typical foci staining pattern of γ-H2AX in both cisplatin and cisplatin/nHNK testis showing cisplatin-induced DNA breaks. (**B**) The level of DNA break was also evaluated in other reproductive organs (i.e., epididymis and seminal vesicles) using. Same γ-H2AX staining procedures were performed in the epididymis and the seminal vesicle; however, DNA break was observed specifically in the testis as no γ-H2AX signal can be detected in the epididymis nor seminal vesicles of the same cisplatin-treated individual. (**C**) Cisplatin significantly reduced the number of sertoli cells, spermatids, but not spermatogonia, and nHNK treatment, despite showed restoration of sertoli cells, but the protective effect was more apparent on rescuing fast dividing spermatids. At least 20 individual images from 5 animals were examined. One-way analysis of variance (ANOVA) with Tukey’s multiple comparisons test was used and statistical difference at *p* < 0.05, N.S.: no statistical different. ** *p* < 0.01; * *p* < 0.05. Representative images were shown.

Higher magnification showed pronounced γ-H2AX signals on spermatogenic layers from cisplatin-treated testis (Figure 6A), this cisplatin-induced DNA breaks in male reproductive tract was specific for testis as both epididymis and seminal vesicles were stained negatively for γ-H2AX (Figure 6B). To elucidate of which testicular cells were affected, quantification analyses were performed by examining spermatogenic cells of different stages. From Figure 6C, we showed that the number of sertoli cells was significantly reduced in cisplatin-treated testis, and nHNK treatment, despite showed protective effects, did not significantly restored the number of sertoli cells (Figure 6C, left panel). Although similar trend was observed for the number of spermatogonia, no significant changes were detected (Figure 6C, middle panel). However, cisplatin induced significant loss of spermatids (both round and elongated spermatids), and nHNK treatment significantly restored the number of spermatids per testicular lumen (Figure 6C, right panel), indicated the most affected spermatogenic cells were fast dividing spermatids rather than sertoli cells or spermatogonia.

### 3.8. nHNK Reduced TGFβ1, TXNDC5, αSMA Protein Expression and Type I Collagen Accumulation Resulted in Minor Testicular Fibrosis

**Figure 7:**
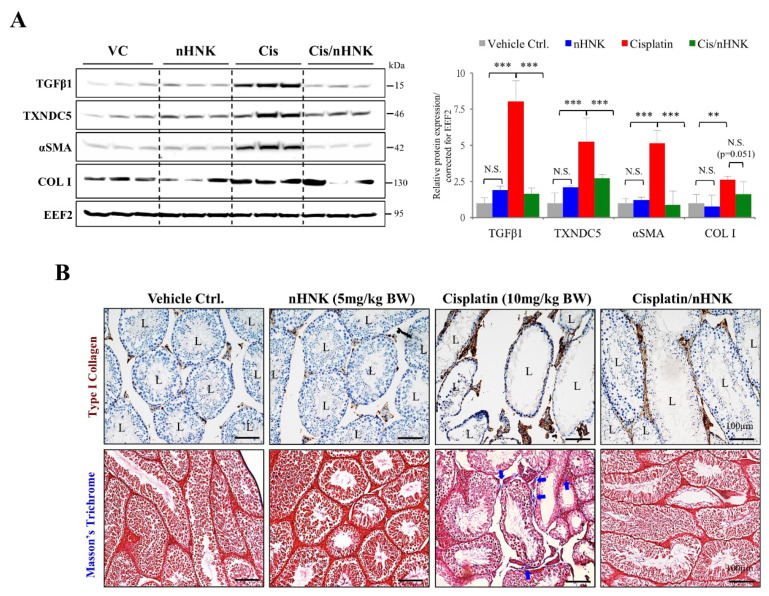
The effects of nanosome honokiol on testicular fibrosis. (**A**) Cisplatin induced TGFβ1 protein expression and nHNK attenuate TGFβ1 activation. Both αSMA and type I collagen protein expression were up-regulated in the testis of cisplatin-injured animals, nHNK greatly reduced the expression levels of both protein expressions. Endoplasmic reticulum protein TXNDC5 was up-regulated by cisplatin administration and nHNK treatment reduced TXNDC5 protein expression in the testis. (**B**) Immunohistochemistry demonstrated excessive accumulation of type I collagen (in brown) at the basal region of the seminiferous tubules and at the interstitial compartment of the testis from cisplatin-treated animals (upper panel). In line with patterns observed for type I collagen, accumulation of collagen stained in blue (indicated with arrows) was apparent when cisplatin-injured testicular section was subjected to trichrome stain (lower panel). VC: vehicle control; nHNK: nanosome honokiol alone; Cis: 10 mg/kg B.W. cisplatin administration; Cis/nHNK: nHNK treatment group. L: lumen. Representative images were shown. One-way analysis of variance (ANOVA) with Tukey’s multiple comparisons test was used and statistical difference at *p* < 0.05, N.S.: no statistical different. *** *p* < 0.001; ** *p* < 0.01. Representative images were shown. L: lumen.

Activation of transforming growth factor beta (TGFβ) is one of the hallmarks for organ fibrosis, downstream activation of alpha smooth muscle actin (αSMA)+ collagen producing cells often results in excessive production, the accumulation of extracellular matrix that consequently lead to organ fibrosis. We showed cisplatin induced up-regulation of TGFβ1 (8-fold increase when compared with control group), αSMA (5.1-fold increase when compared with control group) and type I collagen protein expression (2.6-fold increase when compared with control group) (Figure 7A), these above-mentioned elevations were greatly reduced when nHNK was given as treatment (Figure 7A, compared red bars with green bars). The increased type I collagen protein expression was also evidenced by immunohistochemistry examination and Masson’s trichrome stain, that excessive accumulation of type I collagen was detected in the testis of cisplatin-treated animals, and reduced signals were observed in nHNK-treated group (Figure 7B). Recent study showed ER protein thioredoxin containing domain 5 (TXNDC5) augmented myocardial fibrosis by facilitating extracellular matrix protein folding [38], in line with their findings, we observed that increased TGFβ1, αSMA and type I collagen protein expression was accompanied with significant increased TXNDC5 protein expression (5.3-fold increase when compared with control group), and nHNK treatment led to significantly reduction of TXNDC5 (Figure 7A, Figure S4).

## 4. Discussion

Cisplatin is an effective platinum-containing anti-cancer compound frequently used at clinic; however, severe renal and testicular toxicities are two commonly detected side effects when applied systemically [7,39]. Although other platinum-containing drugs (e.g., carboplatin, oxaplatin) have been developed and used to reduce organ toxicity, these alternative platinum-containing anti-cancer compounds are less potent and exhibit other side effects, such as myelo-supression, neurotoxicity and ototoxicity [40]. Therefore, minimize cisplatin-induced organ toxicity and damages are imminent to improve clinical safety of cisplatin for effective cancer treatments. In this study, we demonstrated that nanosized liposome-encapsulated polyphenol constituent honokiol attenuated cisplatin-induced chronic testicular damages by mitigating testicular ROS production, upregulating mitochondrial anti-oxidation enzyme expressions and downregulating specific BiP-ATF4-CHOP ER stress. The reduction of cellular apoptosis was likely attributed from the minimized DNA oxidative damages and DNA breaks in the testis. Moreover, reduced TGFβ1 and TXNDC5 protein expression led to less αSMA+ fibroblasts activation with reduced type I collagen accumulation at the extracellular matrix of the testis. In line with our observation, Akamata et al., showed HNK blocked intracellular TGFβ signaling and inhibited fibrosis process by direct effects on PKC activity, Sirtuin functions and TGF signaling [41]. It is likely that the alleviation of testicular damages and the reduced fibrosis process by nHNK treatment allowed the restoration of spermatogenic structure, and the subsequent repopulation of spermatozoa in the epididymis.

Besides forming DNA adducts [6], cisplatin also induces excessive ROS production that disrupts physiological balance of redox and anti-oxidation activities [42]. We previously showed both in vitro and In Vivo that cisplatin compromised mitochondria total antioxidant capacity, and as a consequence, led to imbalanced mitochondria redox processes and caspase 3-associated apoptosis [17,18]. Other studies also showed cisplatin induced dose-dependent testicular damage, ROS generation and ER stress in rat testis [10,43,44]. In agreement with these studies, we showed both in vitro and In Vivo that nHNK significantly reduced intracellular ROS production. In Vivo experiments indicated that as low as 5 mg/kg B.W., nHNK can effectively reduce DNA oxidation in both testis and elongated spermatozoa, as significant reduction (45% less) of testicular 8-OHdG signal was observed when nHNK was given as a treatment upon cisplatin injury. Earlier publication from Xu et al. showed that X-ray repair cross-complementation group 1 (*Xrcc1*), a key DNA repair gene, plays a vital role in maintaining genomic stability during early stage spermatogenesis [45]; we showed from RNAseq analysis that cisplatin and HNK also altered *Xrcc1* gene expression (cisplatin down regulated *Xrcc1* gene expression by 3.98 fold when compared with control animal and nHNK upregulated *Xrcc1* gene expression by 3.77 fold, data not showed). This finding likely contributed to the observed defect or restoration of DNA breaks and subsequent affects spermatogenesis.

Mitochondrial enzymes (e.g., glutathione peroxidase, superoxide dismutase, thioredoxin reductase, glutaredoxin, peroxiredoxin) have been correlated with cellular anti-oxidation activity [15,16]; in agreement with our earlier study in the kidney, we did detect significant alterations on gene expression of many above-mentioned mitochondrial anti-oxidation enzymes, suggested testicular mitochondria and their enzymatic anti-oxidation network were affected under our experimental set up.

Testis is an organ characterized with active protein synthesis and folding events due to incessant spermatogenesis and DNA packaging. Moreover, ER stress chaperone Grp78 is permanently present in the pachytene spermatocytes suggesting the prominent role of ER signaling pathway in the testis [46]. Endoplasmic reticulum is required for protein synthesis, folding and modifications, disruption of ER homeostasis leads ER stress and excessive accumulation of unfolded/misfolded proteins [47]. Studies have shown that ROS-induced ER stress activated unfolding protein response (UPR) signaling cascades that impaired endometrial menstrual cycle [47], ovarian folliculogenesis [48], spermatogenesis [49], fertilization, and pre-implantation embryo development [50]. Therefore, excessive ROS generation by cisplatin and the prolonged ER stress likely elicited redox imbalance and disrupted ER homeostasis that eventually lead to the observed testicular damages. One of the bioactivities of honokiol is anti-oxidation ability [21,51], in line with earlier study showed honokiol attenuated torsion-induced testicular damages [19], we demonstrated in this study that nHNK exhibited anti-oxidation effect, and could efficiently reduce cisplatin-induced ROS generation, the reduced ROS likely accounts for the attenuation of Bip-ATF4-CHOP associated ER stress. Despite HNK is effective, we still observed apparent cytotoxicity at higher dose as many other drugs; therefore, usage on caution is still required to ensure the safety of the compound. Lone and Yun showed earlier that HNK reduced ROS production was concurrent with elevation of lipid/fatty acid oxidation related genes (e.g., ACOX1, CPT1, p-HSL, and p-PLIN) [52], and in this study, we focus on HNK effects on reducing protein, DNA oxidation as well as ER stress. Taken together, HNK exhibited broad range ability on modulating fat oxidation, lipid catabolism, DNA and protein oxidation, that result in its ROS scavenging property.

Recent study demonstrated that ER protein TXNDC5 augments myocardial fibrosis by facilitating extracellular protein folding [38], in agreement with this study, we observed that cisplatin up regulation of TXNDC5 was attenuated by nHNK treatment, the reduced type I collagen and (αSMA)+ fibroblasts in the testis likely promoted extracellular protein folding process as described by Shih et al. [38]. However, whether honokiol exhibits direct effect on TXNDC5 gene and protein expression or indirectly reduces ROS and ER stress that lead to the observed changes on TXNDC5 protein expression remained unclear. Liu et al. showed in hepatic stellate cells that increased ECM synthesis and secretion in response to TGFβ stimulation is associated with specific IRE1 signaling-dependent ER stress and UPR [53]. Lenna et al. suggested that ER stress and UPR signaling pathway play a direct profibrotic role in response to TGFβ activation in lung fibrosis [54]. In line with these studies, we showed cisplatin up-regulated TGFβ1 and induced UPR via specific Bip-ATF4-CHOP associated ER stress in the testis, augmentation of ER stress and down regulation of TXNDC5 protein expression by nHNK treatment likely resulted in the reduction of αSMA+ fibroblasts activation and type I collagen deposition in the testis.

## 5. Conclusions

**Figure 8:**
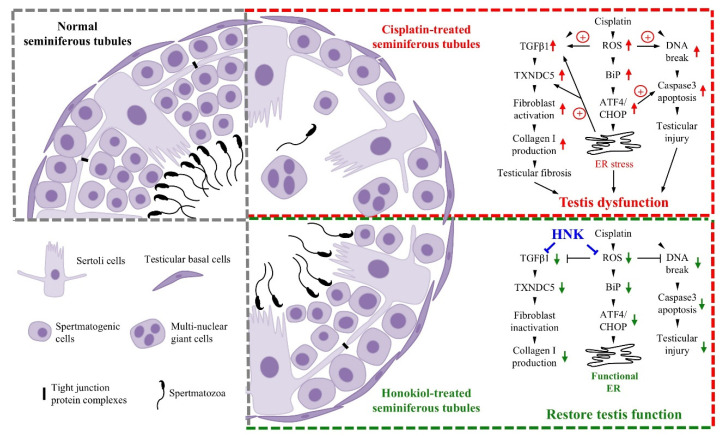
Schematic explanation of cisplatin-induced testicular injury and the counteracting protective effects of nanosome honokiol. Normal testis (in grey box) consists of sertoli cells forming tight junction (black bar) to maintain testicular structure. Multi-layered spermatogenic cells represent normal and ongoing spermatogenesis with different stages of maturating sperm cells. Cisplatin (red box) induces excessive intracellular ROS production that subsequently lead to Bip-ATF4-CHOP ER stress. Additionally, excessive ROS causes DNA breaks in the testis and results in caspase 3-associated cellular apoptosis. Uncontrolled ROS production activates TGFβ1 and upregulates TXNDC5 which subsequently increase αSMA and type I collagen protein expression for fibroblast activation and extracellular matrix accumulation in the testis. All- above mentioned damages result in testis dysfunction and compromise normal sperm production. Nanosome honokiol (in green box) acts as ROS scavenger with its known anti-oxidation bio-property, reduction of cisplatin-induced ROS not only significantly attenuates Bip-ATF4-CHOP ER stress and testicular DNA breaks, but also reduce caspase 3-associated cell death. By down regulation of TGFβ1 and ER protein TXNDC5, fibroblast activation and type I collagen accumulation were both mitigated. Improvement of testicular environment attributes to the restoration of sperm production and normal testicular functions.

In conclusion, we have demonstrated in our current study that liposome-based nanosuspension formulated honokiol provides protective effects against cisplatin-induced chronic testicular damages in vivo. The protective outcomes, the improvement of testicular structure and sperm production likely resulted from the combined effects of (1) reduced ROS generation and Bip-ATF4-CHOP associated ER stress in the testis, (2) reduced DNA breaks in fast dividing spermatids and cellular associated apoptosis, (3) reduced TGFβ1, TXNDC5, αSMA+ fibroblasts activation and type I collagen accumulation at the testicular extracellular matrix; all of the above-mentioned outcomes resulted in improved testicular function and restoration of normal sperm production (graphically summarized in Figure 8). This study provides opportunities to reduce cisplatin-induced testicular damages and might significantly provide benefits in the fertility of patients who undergo cisplatin-based cancer treatments. Our findings render opportunities for re-introducing cisplatin to systemic anti-cancer therapy with significant reduced testicular toxicity, and our results would benefit patients who receive cisplatin-based chemotherapy with restored fertility.

## Figures and Tables

**Table 1:** Testicular damage scoring criteria. A self-defined testis damage scoring system was used to access the level of testicular damage. The scoring was based on the presence of different layers of testicular spermatogenic cells in the seminiferous tubules. The score was given reciprocally to the layers of spermatogenic cells. An additional point was assigned when multinucleated giant cells were observed in the lumen of the seminiferous tubules.

Score	Cellular Layer of the Lumen	Multinucleated Giant Cells
0	>3 layers	additional 1 point
1	2 layers	
2	1 layer	
3	No cell present	

## Supplementary Materials

The following are available online at https://www.mdpi.com/2076-3921/9/8/723/s1, Figure S1: Cytotoxicity evaluation of cisplatin and honokiol. Figure S2 Illustration and validation of cisplatin-induced chronic testicular injury mouse model. Figure S3 Computer assisted sperm analysis (CASA) and acrosome integrity assay for sperm quality evaluation. Figure S4 Original Western-blotting membrane blots. Figure S5 High performance liquid chromatography (HPLC)-MS/MS analysis on mouse tissue samples for the detection of nHNK.
