# Supplementary material for: Counteracting Cisplatin-Induced Testicular Damages by Natural Polyphenol Constituent Honokiol
Source: Antioxidants (Basel). 2020 Aug 9;9(8):723. doi: 10.3390/antiox9080723 (PMC7464045; doi:10.3390/antiox9080723)
Supplement: Supplementary file 1 [file antioxidants-09-00723-s001.pdf]

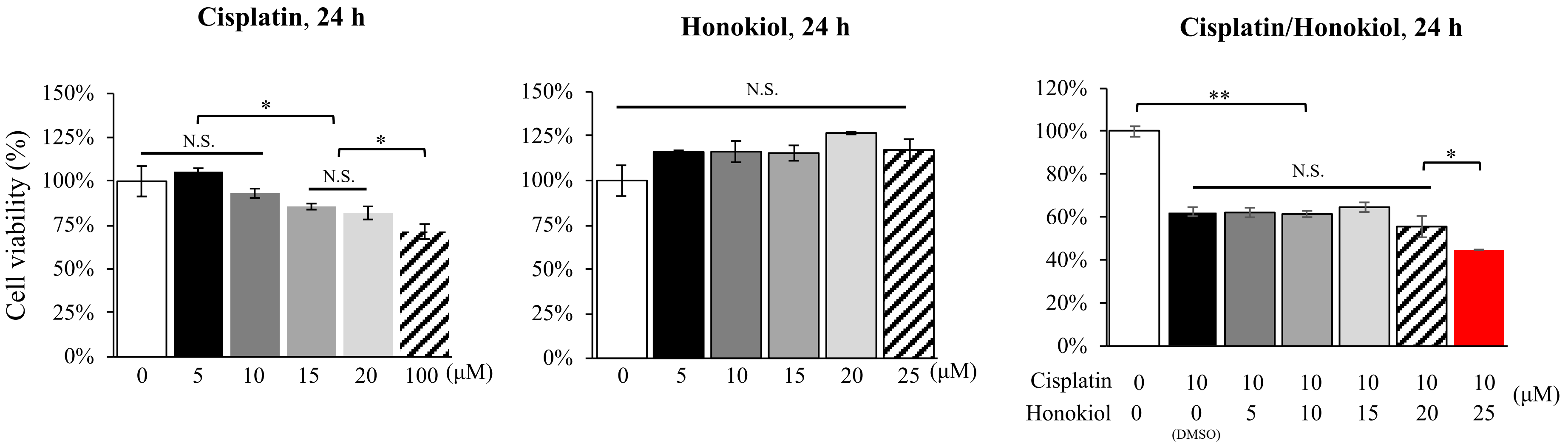

Cisplatin, 24 h
Honokiol, 24 h
Cisplatin/Honokiol, 24 h
Cell viability (%)
150%
125%
100%
75%
50%
25%
0%
N.S.
*
0
5
10
15
20
100
(μM)
25
120%
80%
60%
40%
20%
**
Cisplatin
Honokiol
(DMSO)

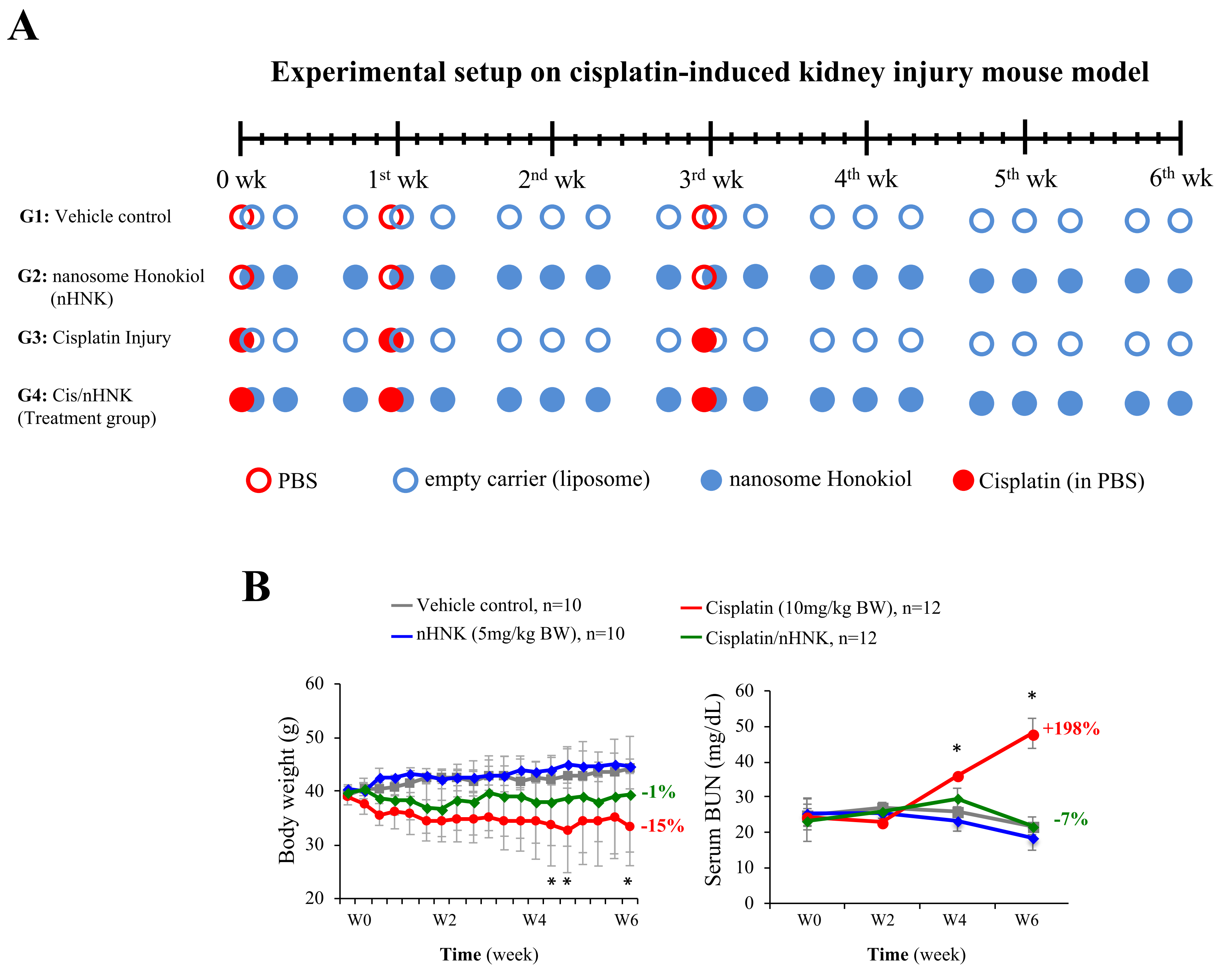
A
Experimental setup on cisplatin-induced kidney injury mouse model
0 wk
1st wk
2nd wk
3rd wk
4th wk
5th wk
6th wk
G1: Vehicle control
G2: nanosome Honokiol (nHNK)
G3: Cisplatin Injury
G4: Cis/nHNK (Treatment group)
PBS
empty carrier (liposome)
nanosome Honokiol
Cisplatin (in PBS)
B
Vehicle control, n=10
nHNK (5mg/kg BW), n=10
Cisplatin (10mg/kg BW), n=12
Cisplatin/nHNK, n=12
Body weight (g)
60
50
40
30
20
W0
W2
W4
W6
-1%
-15%
* *
*
Time (week)
Serum BUN (mg/dL)
60
50
40
30
20
10
0
W0
W2
W4
W6
*
*
+198%
-7%
Time (week)

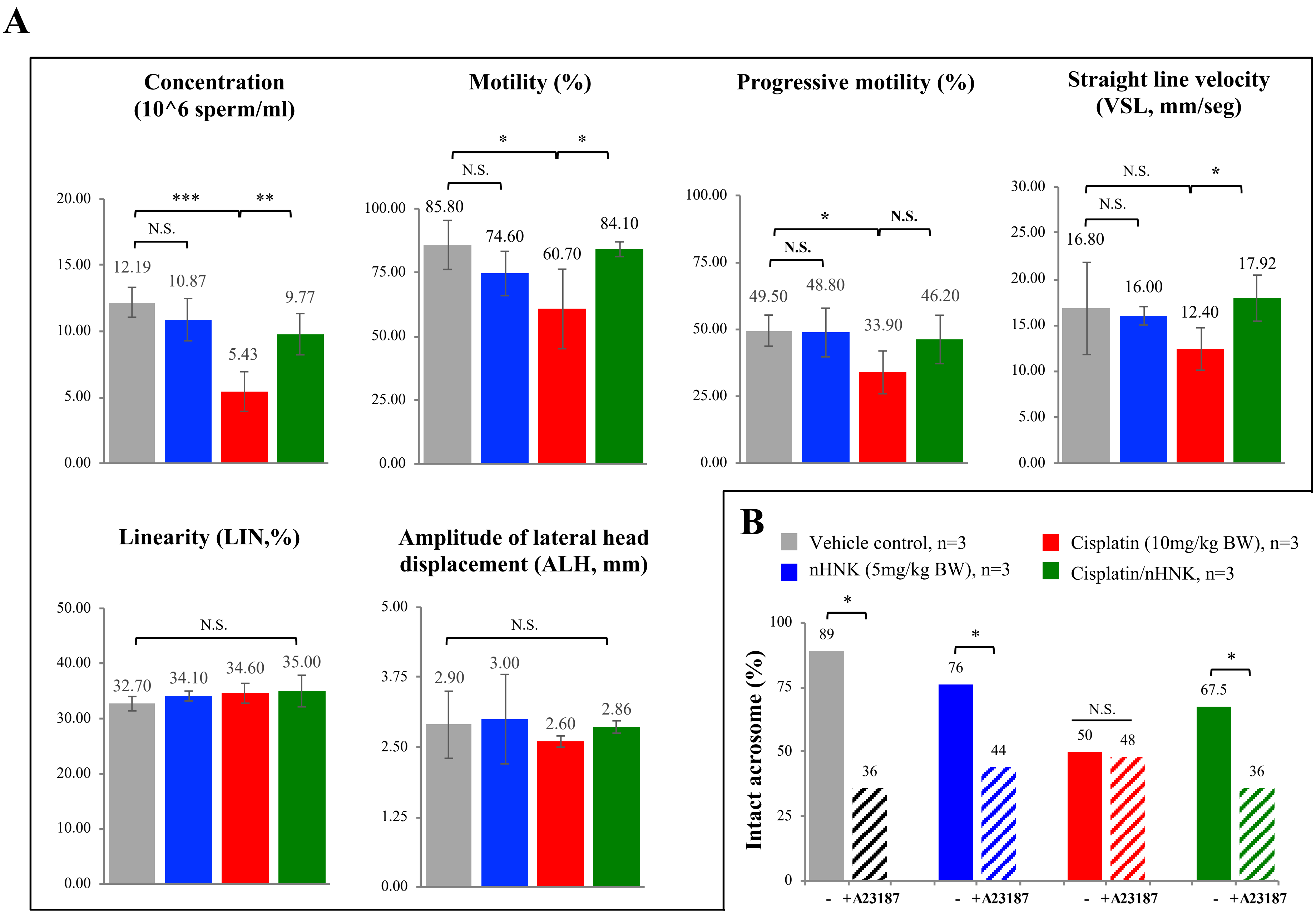
A
Concentration (10^6 sperm/ml)
Motility (%)
Progressive motility (%)
Straight line velocity (VSL, mm/seg)
Linearity (LIN,%)
Amplitude of lateral head displacement (ALH, mm)
12.19
10.87
5.43
9.77
85.80
74.60
60.70
84.10
49.50
48.80
33.90
46.20
16.80
16.00
12.40
17.92
32.70
34.10
34.60
35.00
2.90
3.00
2.60
2.86
N.S.
*
**
***
B
Vehicle control, n=3
nHNK (5mg/kg BW), n=3
Cisplatin (10mg/kg BW), n=3
Cisplatin/nHNK, n=3
Intact acrosome (%)
89
36
76
44
50
48
67.5
36
- +A23187
- +A23187
- +A23187
- +A23187

Fig. 5

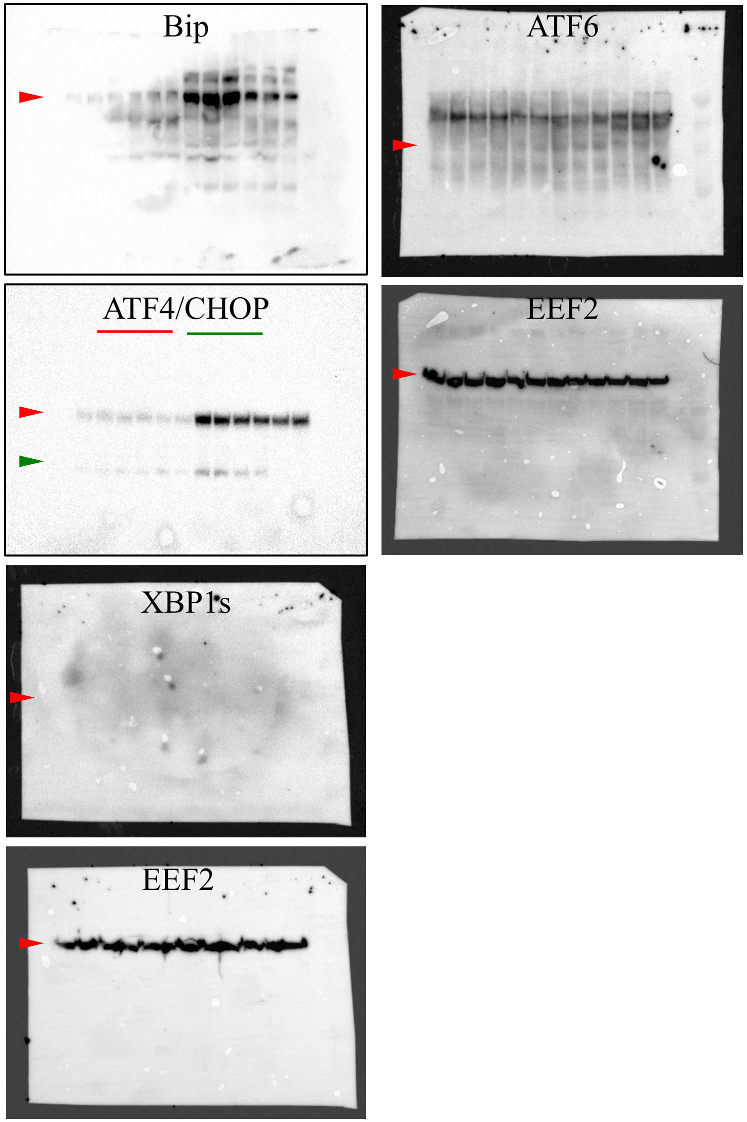


Fig. 6

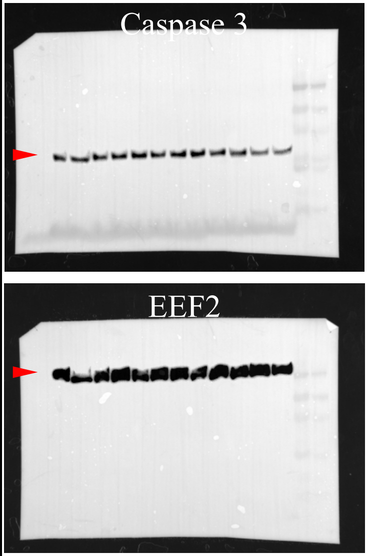


Fig. 7

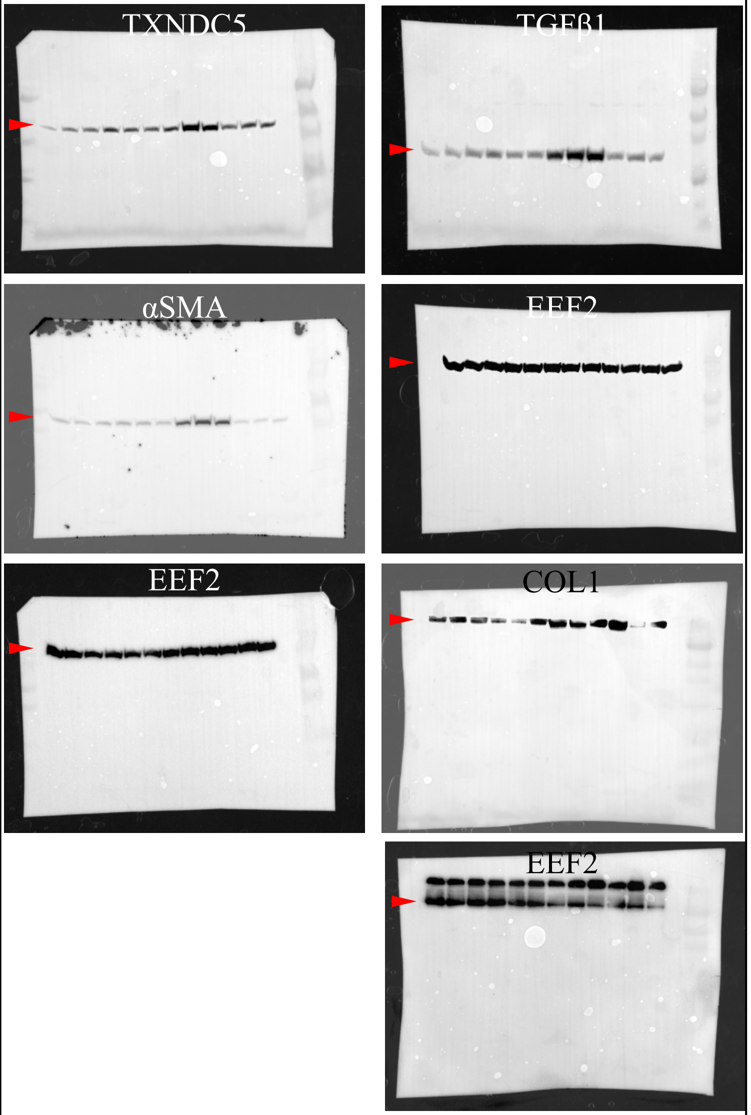

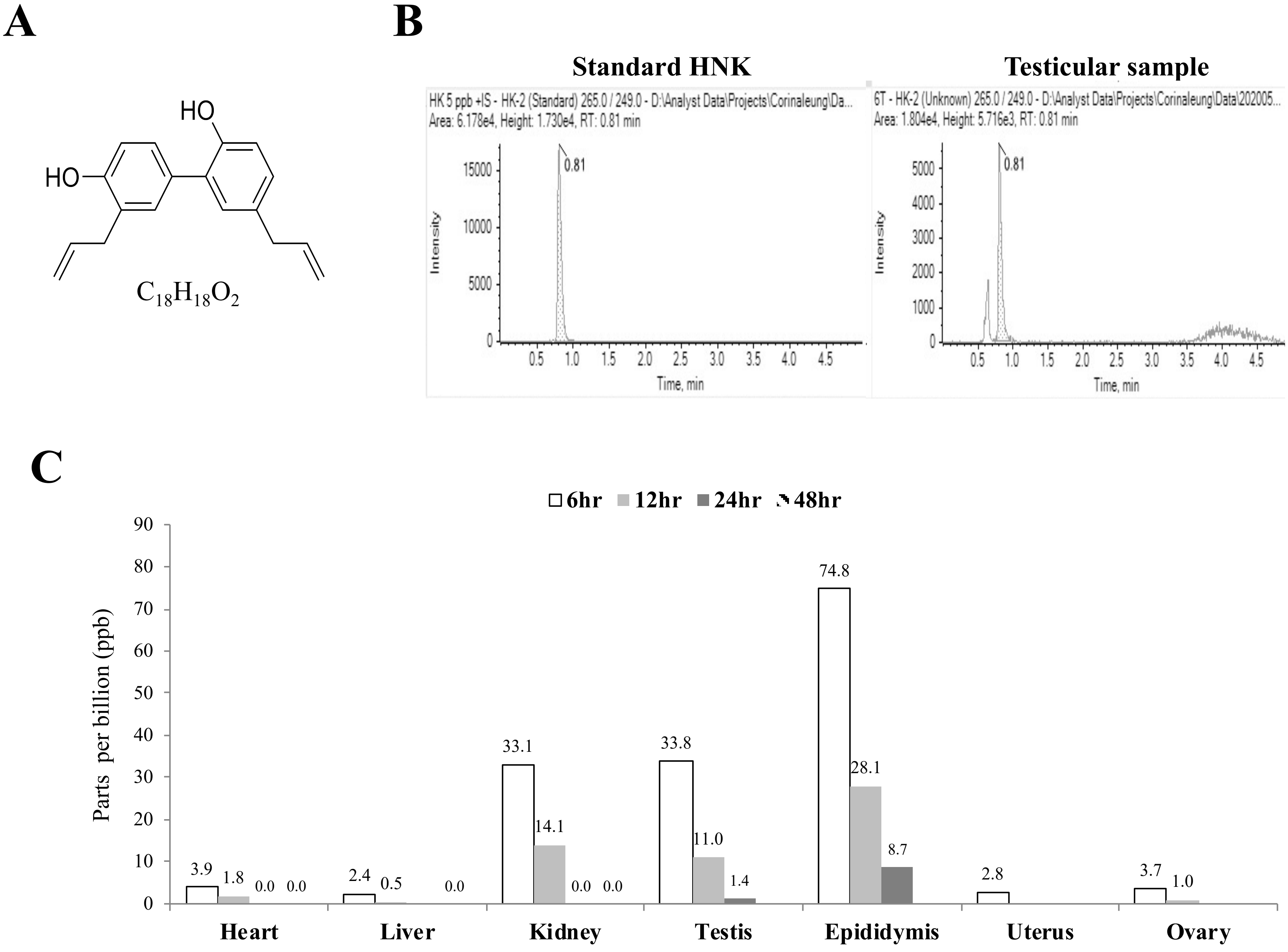
A
HO
HO
C18H18O2
B
Standard HNK
Testicular sample
HK 5 ppb +IS - HK-2 (Standard) 265.0 / 249.0 - D:\Analyst Data\Projects\Corinaleung\Da...
Area: 6.178e4, Height: 1.730e4, RT: 0.81 min
6T - HK-2 (Unknown) 265.0 / 249.0 - D:\Analyst Data\Projects\Corinaleung\Data\202005...
Area: 1.804e4, Height: 5.716e3, RT: 0.81 min
0.81
Intensity
Time, min
C
6hr 12hr 24hr 48hr
Parts per billion (ppb)
Heart
Liver
Kidney
Testis
Epididymis
Uterus
Ovary
